# Ab initio inspired design of ternary boride thin films

**DOI:** 10.1038/s41598-018-27426-w

**Published:** 2018-06-18

**Authors:** Vincent Moraes, Helmut Riedl, Christoph Fuger, Peter Polcik, Hamid Bolvardi, David Holec, P. H. Mayrhofer

**Affiliations:** 1 Christian Doppler Laboratory for Application Oriented Coating Development at the Institute of Materials Science and Technology, TU Wien, A-1060 Wien, Austria; 2 Institute of Materials Science and Technology, TU Wien, A-1060 Wien, Austria; 3 Plansee Composite Materials GmbH, D-86983 Lechbruck am See, Germany; 4 Oerlikon Balzers, Oerlikon Balzers Surface Solutions AG, FL-9496 Balzers, Liechtenstein

## Abstract

The demand to discover new materials is scientifically as well as industrially a continuously present topic, covering all different fields of application. The recent scientific work on thin film materials has shown, that especially for nitride-based protective coatings, computationally-driven understanding and modelling serves as a reliable trend-giver and can be used for target-oriented experiments. In this study, semi-automated density functional theory (DFT) calculations were used, to sweep across transition metal diborides in order to characterize their structure, phase stability and mechanical properties. We show that early transition metal diborides (TiB_2_, VB_2_, etc.) tend to be chemically more stable in the AlB_2_ structure type, whereas late transition metal diborides (WB_2_, ReB_2_, etc.) are preferably stabilized in the W_2_B_5−*x*_ structure type. Closely related, we could prove that point defects such as vacancies significantly influence the phase stability and even can reverse the preference for the AlB_2_ or W_2_B_5−*x*_ structure. Furthermore, investigations on the brittle-ductile behavior of the various diborides reveal, that the metastable structures are more ductile than their stable counterparts (WB_2_, TcB_2_, etc.). To design thin film materials, e.g. ternary or layered systems, this study is important for application oriented coating development to focus experimental studies on the most perspective systems.

## Introduction

Designing new materials is a highly relevant topic covering many different aspects, like discovering materials with entirely new properties (e.g., carbon-nanotubes), improving existing materials in use, (e.g., reduce costs or weight), or increasing biocompatibility or environmental sustainability. These examples are just a small fraction of the never-ending quest fueled by modern industry. The increasing computational power opens new fruitful approaches for materials design^1^. Trial and error can be supported (thus focused) or even replaced by fundamental and sophisticated knowledge-based methods using semi-automated density functional theory calculations to scan across entire material classes^2^. Nitride-based materials, as prototypes of protective thin films for cutting and milling tool applications, have been intensively studied from their binary systems (e.g., TiN, AlN, CrN)^3–5^ up to their ternary^6,7^ or even multinary systems^8,9^. For example, TiN and Ti-Al-N are two highly prominent representatives present widely used in industry (e.g., tooling, microelectronics, decorative purpose). Additionally, considerable research activities concentrate on further application-oriented improvement of these materials using different architectural designs, which will combine their beneficial properties or create entirely new features^10^.

A rather new and extremely promising class of materials–with the potential to be used for many different applications ranging from superconductivity to wear- and corrosion-resistance–are borides^11,12^. Contrary to nitrides, only little is known about borides and more specifically about diborides, with the chemical formula XB_2_ (where X stands for transition metals (TM)). While there are several experimental and theoretical studies on binary diborides^13,14^, multinary diborides are rather unexplored^15–17^. Importantly, ReB_2_ has been theoretically predicted to be the most incompressible material exceeding the properties of diamond suggesting the use in (iron alloys) cutting applications were traditional materials (e.g., diamond) cannot be used due to the formation of carbides^18,19^. A huge drawback, when consindering TM-diborides for hard coating applications is the pronounced brittle behaviour of this material class. Hence, it is of great importance to classify their ductility and increase the toughness.

**Figure 1:**
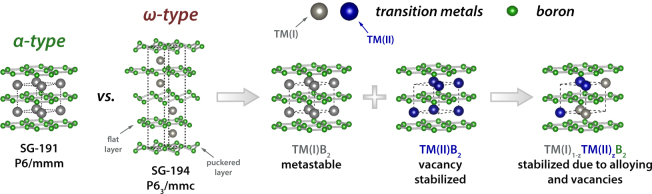
Competing structure types of transition metal diborides (*α*, space group 191, P6/mmm, vs. *ω*, space group 194, P6_3_/mmc)^2,20^ and possible phase stabilizing routes for AlB_2_ structured diborides by alloying of a second transition metal and/or point defects (ternary boride).

Many diborides tend to crystallize in two different hexagonal structures. While early transition metal diborides crystallize in the so-called AlB_2_ structure type (*α*, space group 191 - P6/mmm)^2^, late transition metal diborides (e.g., Tungstendiboride) prefer a W_2_B_5−*x*_ based structure (*ω*, space group 194 - P6_3_/mmc)^20^ - see Fig. 1. The AlB_2_ *α*-structure consists of a hexagonal shaped unit cell with an alternating stacking of covalently bonded graphite-like boron hexagons and metal layers. The closely related W_2_B_5−*x*_ *ω*-structure type consists of hexagonal unit cell with alternating flat and puckered boron hexagons between the metal layers. Regarding the synthesis via physical vapor deposition, it is a well known fact, that metastable structures can be captured via this synthesis route^21,22^, which is often achieved by the implementation of point defects such as vacancies^23,24^. This circumstance of depositing a metastable structure can lead to extraordinary and positive effects (e.g., AlN, which shows significantly higher mechanical strength and elastic constants for its metastable face centered cubic structure than its thermodynamically stable hexagonal close packed wurtzite-type structure) but includes also the fact that upon providing the necessary activation energy, the metastable structure will transform in its thermodynamically stable modification (which is well known and investigated for many of the metastable Al_2_O_3_ polymorphs that transform to corundum-type Al_2_O_3_). Here, materials science allows for at least two helpful scenarios: 1) decreasing the difference in Gibbs free energy between metastable and stable state (which decreases the driving force for the phase transformation) and 2) increasing the Gibbs free energy of the peak separating the metastable and stable state (which increases the necessary activation energy to reach the stable state.) Picking up the concept of ZrO_2_ based ceramics, where alloying of certain elements (e.g., Mg, Y, Ce)^25^ leads to stabilization of high temperature modifications at room temperature, the two hexagonal diboride phases draw a great starting point for the exploration of ternary diboride materials. The combination of these phase stabilizing routes for the preferred *α*-type structure are schematically depicted in Fig. 1. To use such concepts for designing new materials (not necessarily limited only to thin films), it is essential to know and understand the fundamental properties (e.g., energy of formation (E_*f*_), lattice constants, etc.) of the building blocks. Furthermore, such a knowledge database allows to set-up target-oriented experiments aiming on fulfilling various demands.

In this study, we use density functional theory (DFT) to obtain basic properties of transition metal diborides (TMB_2_) in their AlB_2_ and W_2_B_5−*x*_ structures (*α*-TMB_2_ and *ω*-TMB_2_). In addition to perfect structures we also investigate the impact of vacancies on the energy of formation (which characterizes the chemical stability) as materials prepared by physical vapor deposition techniques are known for their high density of point defects such as vacancies. Since mechanical properties are of central importance for protective coatings, we calculated the elastic constants (bulk and shear modulus, poisson ratio, and the cauchy pressure) of all these transition metal diborides (3 to 5d elements excluding Hg) in their *α* and *ω* crystal structure.

## Results and Discussion

### Ground state properties

**Figure 2:**
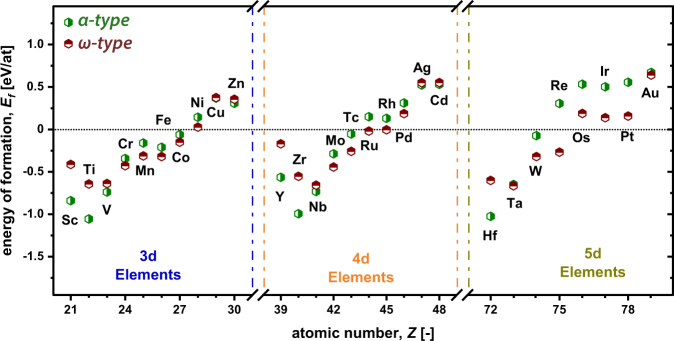
Energies of formation (E_*f*_) per atom for all TMB_2_ investigated (3d, 4d, and 5d transition metal elements, excluding Hg). The green and red hexagons represent the *α*- (AlB_2_ prototype) and *ω*- (W_2_B_5−*x*_ Prototype) type structures, respectively. Please not, that some diborides have very similar *E*_*f*_ in the two structure types and therefore overlapping symbols (e.g. Cu or Ta).

The energy of formation E_*f*_, which is a fundamental indicator (for chemical stability) of a solid matter, quantifies the energy gain (for negative E_*f*_ values) upon forming a compound out of the individual elements, calculated as:

1 $${E}_{f}=\frac{1}{{\sum }_{i}{n}_{i}}({E}_{tot}-\sum _{i}\,{n}_{i}{E}_{i})$$

where, E_*tot*_ is the total energy of the compound (here, TMB_2_), E_*i*_ the energy of the individual elemental constituent *i* in its stable crystalline configuration, and n_*i*_ denotes the number of atoms for the different species *i*. Figure 2 shows the energy of formation for all 28 TMB_2_ materials calculated, spanning the whole range of 3d, 4d and 5d (excluding Hg) transition metals, in the two different but relevant hexagonal structures, *α*- and *ω*-type.

With increasing atomic number (within each period), the E_*f*_ values become less negative and even positive (i.e., chemically instable) for TMB_2_ with Ni, Cu, Zn (3d elements), Pd, Ag, Cd (4d elements), Os, Ir, Pt, Au (5d elements). RuB_2_ and RhB_2_ (4d elements) are special border candidates as E_*f*_ is negative for their *ω*-structure but positive for their *α*-structure. The increasing chemical stability (i.e., more negative E_*f*_ values) with decreasing atomic mass (within the individual periods) underlines the increased reactivity of atoms with fewer valence electrons. Scandium and Yttrium slightly deviate from this tendency, which is comparable to the results obtained for nitrides^26^.

The data also show that the early TMB_2_ prefer the *α*-structure, which is in good agreement with earlier reports^16^, and the largest difference in E_*f*_ between the *α* and *ω* structure of chemical stable TMB_2_ is obtained for TiB_2_, ZrB_2_, and HfB_2_, respectively. These results agree with the density of states (DOS), which show a pseudogap between the bonding and antibonding (TM-d and B-p) states. Within each period, the occupied bonding states change, to partially filled anti-bonding states with increasing atomic number. The decreasing N(E_*F*_) (number of states at the fermi level (E_*F*_) - corresponding to the position of the fermi level) reflects the chemical stability^27^ and is in excellent agreement with literature reports^28–30^. Hence, in the case of *α*-structured TiB_2_, ZrB_2_, and HfB_2_ the fermi level can be found in the pseudogap (lowest N(E_*F*_) - showing highest chemical stability), whereas for ScB_2_ and YB_2_ as well as VB_2_, NbB_2_, and TaB_2_ the fermi level can be found in the bonding or antibonding states, respectively (reduced chemical stability). Furthermore, this circumstance also reflects the trend in melting temperatures (and the directionality of the bonding - covalent bonding character) for the individual diborides^31,32^.

**Figure 3:**
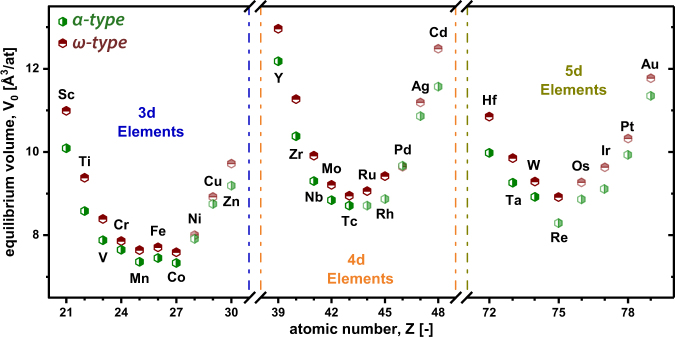
Equilibrium volumes for all TMB_2_ obtained from fitting the Birch-Murnaghan equation. Green and red hexagons denote to euqilibrium volumes for *α*- and *ω* type, respectively. The elements showing positive E_*f*_ (see Fig. 2) are faded - starting at Ni, Ru, and Os for 3d, 4d, and 5d elements, respectively.

As expected from the presence of puckered B-planes in the W_2_B_5−*x*_ structures, all *ω*-TMB_2_ exhibit a higher equilibrium volume (and therefore lower mass density, which is not shown here) as compared to their corresponding *α*-TMB_2_ relatives, see Fig. 3. The equilibrium volume decreases with the atomic radii (in each period), but only up to that transition metal where E_*f*_ becomes positive, please compare Figs 2 and 3. For higher atomic radii of the TM, the euquilibrium volume of the TMB_2_ increases again.

**Figure 4:**
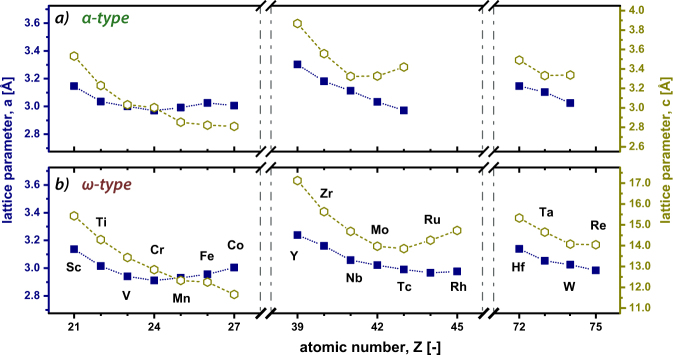
Lattice parameters *a* and *c* for all diborides studied, obtained from fitting the Birch-Murnaghan equation for stable structures (negative E_*f*_). In (**a**) the lattice parameter for the *α*- and in (**b**) those for the *ω*-type structure are given.

In respect to the lattice parameters (shown in Fig. 4a,b) *a* and *c*, and their ratio (*c*/*a*), the trends are consistent for both structure types. For elements from the 3d period, lattice parameter *a* stays fairly constant whereas lattice parameter *c* draws a clear decrease. Therefore, the trend for the c/a-ratio is dominated by the change in *c* with increasing atomic number. Elements from the fourth period show a decrease in lattice constants *a* and *c* until MoB_2_ for the *α*-structure and TcB_2_ for the *ω*-structure. Hence, the c/a-ratio for the *α*-structure decreases to the point where the compounds energetically prefer the *ω*-structure. For the 5d elements both, the *a* and *c* lattice parameter, slightly decrease with increasing atomic number. *α*-VB_2_ as well as *α*-CrB_2_ even reveal a cubic-like c/a-ratio of ~1. Farenholtz *et al*.^33^ stated, that the highly covalent B-B bonds are strong compared to TM-B or TM-TM bonds, which explains the small changes in lattice parameter a, with increasing atomic number (especially for the *α*-type). On the contrary, lattice parameter c is more dominated by the different atomic radii of the specific TM. Furthermore, stable *α*-TMB_2_ are only formed with respect to strained B-B bonds (increased/decreased bonding length caused by the present TMs), which would be crucial for smaller TM-atoms as Cr or larger than Zr. The trends for the *ω*-type is a rather controversial discussed topic in literature and difficult to relate to experimental data. Several experimental and computational studies have been conducted treating the off-stoichiometry of this crystal structure (W_2_B_5−*x*_-prototype)^34,35^. Nevertheless, assuming perfect structures, the observed trends follow similar behavior as obtained for the *α*-type, which leads to the conclusion, that the presence of the puckered boron planes compensate the strain introduced by other TM-atoms such as Mo or W.

### Mechanical properties

Outstanding mechanical properties, e.g. super- or ultra-hardness, strongly correlate to bonding types and hence strength. Fitting the total energy data obtained for variable volumes, with the Birch-Murnaghan equation of state, gives the bulk moduli quantifying the materials’ resistance to isotropic pressure, and therefore represents a good guide for mechanical properties. The data (see Appendix S.1 and S.2) exhibit a maximum for each period as a function of the atomic number, which is at *α*-CrB_2_ (B = 287 GPa), *α*-MoB_2_ (B = 307 GPa), and *ω*-ReB_2_ (B = 333 GPa), for the 3d, 4d, and 5d elements, respectively. Also within our study, ReB_2_ exhibits the highest bulk modulus among all TMB_2_ studied. This is in excellent agreement with literature, stating that ReB_2_ is one of the hardest material available, approaching or even exceeding the excellent properties of diamond^18,19^. Comparing the maxima with the equilibrium volumes, their lowest volumes are at slightly higher atomic numbers, but within the chemically stable regions. As these ceramic-like TMB_2_ materials exhibit a mixture of metallic, covalent, and ionic bonds, the bulk moduli maxima (which are within the chemically stable region, as mentioned) are an indication of stronger bonds leading also to smaller interatomic distances (represented by the specific volume). Generally, the *α*-TMB_2_ exhibit higher bulk moduli than their *ω*-TMB_2_ counterparts, corresponding to their smaller specific volumes, only MnB_2_, TcB_2_, and WB_2_, slightly deviate, and FeB_2_ strongly deviates from this trend.

The entire elastic tensor was calculated for all TMB_2_ compounds in both structures, *α* and *ω*. These allow evaluating semi-empirical criteria for the ductile behavior. In this study, we use three different criteria: the Cauchy pressure (being C_12_–C_44_), the Pugh criteria (*G*/*B* ratio) as well as Frantsevich criteria on the Poisson ratio. After Pettifor *et al*.^36^, a positive Cauchy pressure indicates a metallic bonding character, hence a ductile behavior. Consequently, a negative Cauchy pressure indicates a brittle behavior due to the directional bonding character (hence more covalent contribution). Frantsevich^37,38^ and Pugh^39^ introduced additional criteria to classify a brittle or ductile behavior, by using the Poisson ratio (*ν*) and the *G*/*B* ratio, respectively. A ductile behavior is obtained for *ν* > 0.26 (Frantsevich criterion) or for *G*/*B* < 0.57 (Pugh criterion).

**Figure 5:**
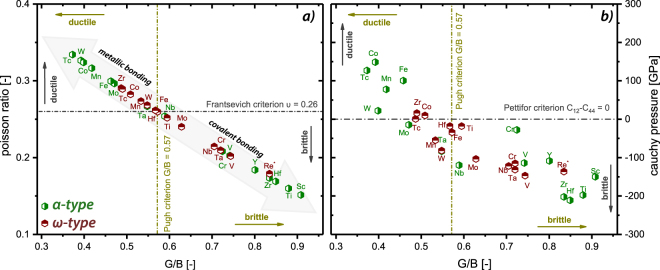
Pugh criterion (*G*/*B*-ratio) as a dependence of the Poisson ratio (Frantsevich criterion, (**a**)) and the Cauchy pressure (C_12_–C_44_), Pettifor criterion, (**b**). The green and red hexagons correspond to *α*- and *ω*-structure, respectively. (*ReB_2_ in **a,b**) was taken from literature^50^. Please note, that some diborides have very similar values and are therefore overlapping.

After these three criteria, presented in Fig. 5a (Frantsevich vs. Pugh criterion) and Fig. 5b (Pettifor vs. Pugh criterion), the most ductile *ω*-TMB_2_ compounds are ZnB_2_, PdB_2_, NiB_2_, and AuB_2_, but these are actually chemically not stable (positive E_*f*_ and therefore not plotted in Fig. 5). All other *ω*-TMB_2_ compounds are at the minimum of at least one criterion or are classified as brittle. Please note that within Fig. 5a,b not all data points are shown, namely *ω* type ScB_2_, YB_2_, RuB_2_, as well as RhB_2_, suggesting either extreme brittle or non-stable structures (more positive E_*f*_ as compared to their *α* type counterpart). The classical stable *α*-TMB_2_, which exhibit a more negative E_*f*_ value than their *ω*-TMB_2_ relatives - ScB_2_, YB_2_, TiB_2_, ZrB_2_, HfB_2_, NbB_2_ - are classified as brittle after all three criteria. Contrary to that, there are many chemically stable *α*-TMB_2_ compounds, like MnB_2_, FeB_2_, CoB_2_ (3d), and TcB_2_ (4d), and WB_2_ (5d), which are classified as ductile after all three criteria. Also *α*-MoB_2_ as well as *α*-TaB_2_ can be added to this list as it is ductile after Frantsevich and Pugh and only slightly brittle after Pettifor. These transition metal diborides actually prefer the *ω*-structure, but are believed to be stabilized in their *α*-structure by introducing point defects such as vacancies (see following sections). Here, the energetically barrier is very small for *α*-TaB_2_, see Fig. 2.

Recalling Fig. 2, we see that the chemical stability (increases with decreased N(E_*F*_)) is reflected in the brittle/ductile-behavior of the individual compounds. Liu *et al*. recently showed by studying differnt structure types for WB_2_, that *α*-structured WB_2_ reveals smaller number of bonds accompanied by increased bond lengths. Therefore, *α*-WB_2_ is predicted to obtain a decreased hardness compared to its *ω*-structured polymorph. This is in good agreement to our results stating that *α*-WB_2_ shows a rather metallic bonding character than in its *ω*-structure, hence being in the highly ductile regime after all criteria.

### Influence of vacancies on the phase stability of TMB_2_

Because PVD promotes the formation of many point defects such as vacancies–and these mutually influence not just the preferred structure^28^, mechanical properties^40^, thermal properties^5^, or magnetic properties^41^, for example–the impact of TM as well as B vacancies on the phase stabilities of *α*-TMB_2_ and *ω*-TMB_2_ is studied. To optimize the supercell size, which should be large enough (to allow the calculation of low vacancy concentrations and to minimize their mutual interaction), supercells with 3 × 3 × 3 (81 lattice sites) and 3 × 3 × 1 (with 108 lattice sites) were used for *α*-TMB_2_ and *ω*-TMB_2_, respectively. Vacancies in these structures were created by removing either one boron or one TM atom, hence ~3.7 at.% TM and ~1.9 at.% B vacancies in *α*-TMB_2_, and ~2.8 at.% TM and ~1.5 at.% B vacancies in *ω*-TMB_2_.

**Figure 6:**
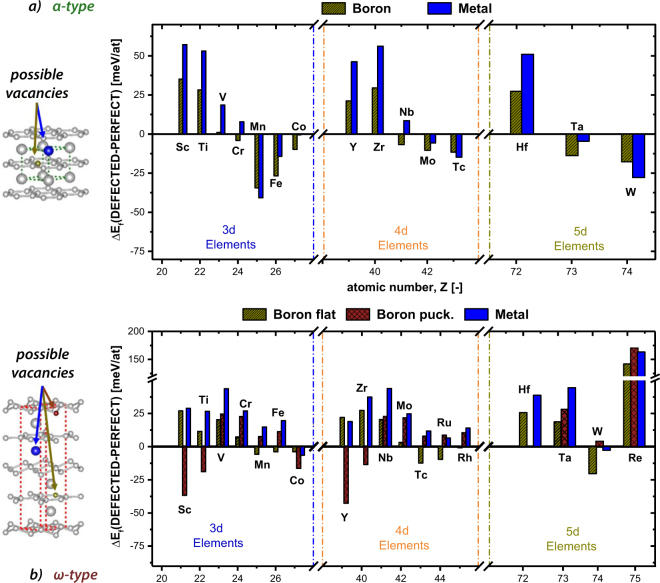
(**a**) Energies of formation per vacancy for all TMB_2_ crystallizing in *α* structured cells. The yellow hatched bars indicate the energies of formation of a randomly taken boron vacancy. The blue bars represent the values for a randomly taken metal vacancy. (**b**) Energies of formation per vacancy for all TMB_2_ crystallizing in *ω*. The yellow and red hatched bars indicate the energies of formation of a randomly taken boron vacancy on the flat and puckered planes. The blue bars represent the values for a randomly taken metal vacancy.

For the *ω*-TMB_2_ structure, the effect of B vacancies is studied for the flat and the puckered B plane individually. The energies of formation for such defected structures (${{\rm{E}}}_{f}^{vac}$), also calculated using Equation 1, are used to obtain the difference in E_*f*_ (ΔE_*f*_  = ${{\rm{E}}}_{f}^{vac}$ − ${{\rm{E}}}_{f}^{perf}$), presented in Fig. 6. Thus, if ΔE_*f*_ is positive, the formation of vacancies is energetically not preferred. Many of the *α*-TMB_2_ compounds actually prefer the formation of vacancies, such as CrB_2_, MnB_2_, FeB_2_, CoB_2_ (3d), and MoB_2_, TcB_2_ (4d), and TaB_2_, WB_2_ (5d). Whereas CrB_2_ and NbB_2_ only prefer the formation of B vacancies, the other mentioned *α*-TMB_2_ compounds prefer the formation of B as well as TM vacancies. Comparing Fig. 6a with Fig. 2 clearly shows that those *α*-TMB_2_ compounds, which are metastable with respect to *ω*-TMB_2_ (e.g. MnB_2_, MoB_2_, WB_2_), favour the formation of vacancies, whereas those *α*-TMB_2_ compounds, which are stable with respect to *ω*-TMB_2_ do not want to have vacancies (e.g. TiB_2_, ZrB_2_, HfB_2_). Although ΔE_*f*_ is also negative for NiB_2_ (3d), and RuB_2_, RhB_2_, PdB_2_ (4d), and OsB_2_, IrB_2_, PtB_2_, AuB_2_ (5d), these compounds still exhibit positive ${{\rm{E}}}_{f}^{vac}$ values, and are thus still chemically instable.

The majority of the TMB_2_ compounds are chemically destabilized by vacancies in their *ω*-structure. Although ΔE_*f*_ is negative for CuB_2_, CdB_2_, OsB_2_, and AuB_2_, these compounds are still chemically unstable with ${{\rm{E}}}_{f}^{vac}$ being positive. ScB_2_, TiB_2_ (3d), and YB_2_, ZrB_2_ (4d), are interesting compounds, as the formation of B-vacancies is only preferred at the puckerd planes, whereas for MnB_2_, FeB_2_ (3d), TcB_2_, RuB_2_, (4d), and WB_2_ (5d), the formation of B-vacancies is only preferred at the flat planes.

If we consider those TMB_2_ compounds that exhibit a metastale *α*-TMB_2_ (with respect to *ω*-TMB_2_) without defects, the preference for *ω*-TMB_2_ or *α* can alter when introducing vacancies. In this respect, we only concentrate on those TMB_2_ compounds that are chemically stable (i.e., negative E_*f*_ values), even when introducing defects (i.e., still negative ${{\rm{E}}}_{f}^{vac}$ values), which are CrB_2_, MnB_2_, FeB_2_, CoB_2_ (3d), and MoB_2_, TcB_2_(4d), and TaB_2_, WB_2_ (5d). As for these compounds ΔE_*f*_ is always more negative for the *α*- than for the *ω*-structure, the two structures come closer in E_*f*_ with introducing vacancies. For TaB_2_, already the 3.7 at.% TM and 1.9 at.% B vacancies considered in this study, the *α*-structure overrules the *ω*-structure. For 2.8 at.% TM vacancies or 1.4 at.% B vacancies, *ω*-WB_2_ is still slightly preferred over the *α*-WB_2_ relative, but the difference significantly decreases from ~25 meV/at (no vacancies) to ~21 meV/at. Thus, we propose that the defects induced by PVD processes are responsible for the preferred formation of *α*-WB_2_^33,34^ rather than the chemically more stable *ω*-WB_2_, if no defects are considered.

### Designing ternary TM(I)_1−*x*_TM(II)_*x*_B_2_ thin film materials

**Figure 7:**
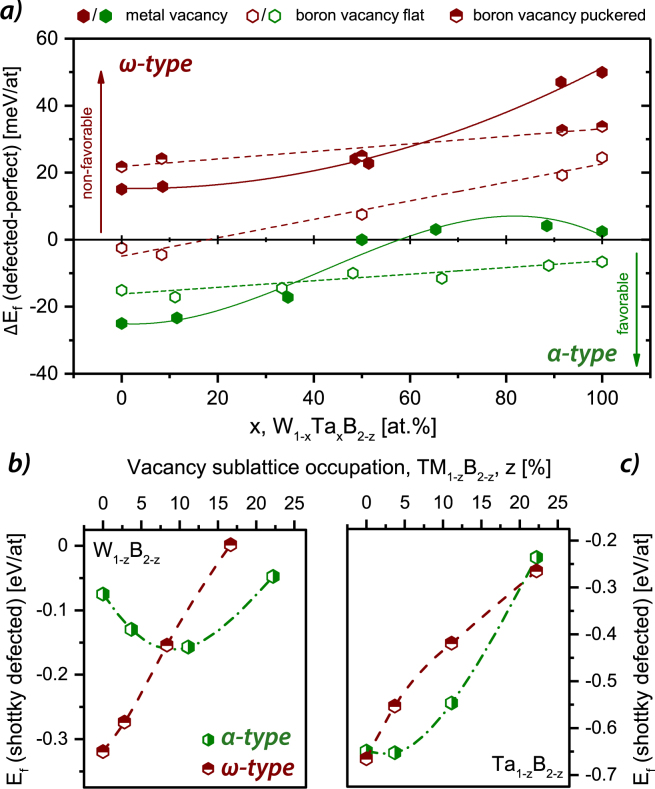
Impact of metal and boron (flat (open symbols) and puckered (half filled symbols) vacancies on the stability of *α*- and *ω*-structured W_1−*x*_Ta_*x*_B_2−*z*_ depending on the Ta/(Ta + W) ratio (**a**). Impact of vacancy concentration (shottky defects) on E_*f*_ of the two discussed structures for WB_2_ (**b**) and TaB_2_ (**c**). The green and dark red symbols correspond to the *α*- and *ω*-structure, respectively.

Several experimental studies on WB_2_ report the formation of the *α*-structure when deposited as thin film via physical vapor deposition^16,42,43^. Taking into account the results presented before, where *α*-WB_2_ is located in the ductile regime (for all criteria), this compound is the ideal candidate for designing ternary borides. Furthermore, finding a good alloying element â€“ with respect to matching lattice constants (equilibrium volume), high bulk modulus, similar tendency for vacancies, and lower E_*f*_ - TaB_2_ points out as an excellent candidate for stabilizing the structure with low cost on ductility. Figure 7, shows the vacancy impact on the *α*- and *ω*-structure in the ternary W_1−*x*_Ta_*x*_B_2−*z*_ system (see Fig. 7a) and the impact of shottky (stoichiometric) defects on the binary systems (see Fig. 7b,c). Clearly, the data for the *α*-structure reveals the favor in stabilizing the structure via vacancies decreasing with increasing Ta content. Contrary to low Ta contents, where the structure strongly desires metal defects, the structure even gets slightly destabilized at high Ta contents for metal vacancies. The small discrepancy to the data presented in Fig. 6 is based on the uncertainty (in the range of 1 to 2 meV/at) of the calculations and the use of different SQS supercells. However, B vacancies stabilize in the full compositional range the *α* structure (also negative E_*f*_ for all compositions investigated). Especially for *α*-TaB_2_, this is in good agreement to experimental studies showing a slight boron deficiency^44^. For *α*-WB_2_, no experimental studies regarding the chemical composition have been conducted so far.

For the *ω*-structure the trends for increasing Ta contents remain, whereas the different vacancy types destabilize the structure. Exceptions here are boron vacancies (flat layer) at high W contents where a (insignificant) gain in energy can be obtained. Considering the results for shottky defects (see Fig. 7b,c) introduced in the *α*- and *ω*-structure for the binaries, it can be clearly seen, that the *α*-structure is more stable at ≥7.5 at.% vacancies and in the range of 0 > *x* ≥ 22.5 at.% for WB_2_ and TaB_2_, respectively. In respect to the concept shown in Fig. 1, the data suggests to start experimental work on W_1−*x*_Ta_*x*_B_2_ thin films. These films should be strongly stabilized due to alloying (low Ta content) and the implementation of vacancies. Furthermore, the material should show a rather ductile behavior compared to “classical” diborides like TiB_2_. Preliminary experimental studies on single-phased DC magnetron sputtered *α*-W_1−*x*_Ta_*x*_B_2_ (with x = 0, 0.07, 0.14, 0.26) thin films showed, that by annealing in vacuum the decomposition of the *α*-phase is postponed with increasing Ta content from 800–1000 °C (*α*-WB_2_) to 1200–1400 °C (*α*-W_0.74_Ta_0.26_B_2_). Additionally, micromechanical bending tests of cantilevers revealed a small decrease in fracture toughness for increasing Ta content from ~$3.7\,MPa\sqrt{m}$ (*α*-WB_2_) to ~$3.0\,MPa\sqrt{m}$ (*α*-W_0.74_Ta_0.26_B_2_).

## Conclusion

Summarizing the results, we have confirmed the tendency of early TMB_2_ compounds preferring the *α*-structure due to their lower E_*f*_ values compared to the *ω*-structure. TMB_2_ compounds with higher atomic numbers in their period than VB_2_ (3d), NbB_2_ (4d), and HfB_2_ (5d) reveal more chemical stability in the *ω*-structure.

The equilibrium volume of the individual elements in the *α*-structure is throughout smaller compared to their *ω*-structured counterpart due to the absence of the puckered boron planes. Moreover, it decreases with atomic radii up to that transition metal where E_*f*_ becomes positive. Regarding the bulk moduli, the opposite trend compared to the equilibrium volume is shown reaching its maxima for *α*-CrB_2_ (3d), *α*-MoB_2_ (4d), and *α*-ReB_2_ (5d). Based on our studies on point defects, we can conclude, that diborides where the *α*-structure is chemically more stable than the *ω*-structure are destabilized when introducing metal or boron vacancies. Furthermore, ScB_2_, TiB_2_, YB_2_, and ZrB_2_ prefer the formation of vacancies on the puckered boron plane when considering the *ω*-structure which underlines their strong tendency to crystallize in the *α*-structure.

After applying three different criteria (Frantsevich, Pugh, and Pettifor) on the mechanical properties obtained from the calculation of the full elastic tensor, *α*-MnB_2_, *α*-FeB_2_, *α*-CoB_2_, *α*-TcB_2_, and *α*-WB_2_ are classified as ductile diborides. Hence, these are all TMB_2_ in the *α*-structure, where the *ω*-structure shows more chemical stability due to the more negative E_*f*_ value.

Based on our calculations we propose *α*-W_1−*x*_Ta_*x*_B_2−*z*_ as a promising ternary material system, providing excellent mechanical strength as well as ductility. WB_2_ only provides excellent ductility data (according to the three ductility criteria) when stabilized in the metastable *α*-structure (AlB_2_-type). With the addition of vacancies (which are typical for physical vapor deposited materials) the *α*-structure becomes energetically preferred over the *ω*-structure (W_2_B_5−*x*_ type). TaB_2_ exhibits almost the same energy of formation for the *α*-as well as *ω*-structure, and again significantly better ductility data for the *α*-structure. Only the Pettifor criterion indicates *α*-TaB_2_ as brittle whereas the other two criteria (Pugh and Frantsevich) indicate *α*-TaB_2_ as ductile. Thus, the addition of Ta is predicted to further promote the stabilization of *α*-WB_2_ structure with relatively low costs in ductility.

## Methods

Applying density functional theory (DFT) coded in VASP (Vienna Ab Initio Simulation Package)^45,46^ using the projector augmented waves method within the generalized gradient approximation (PAW-PBE)^47^, structure and stability of transition metal diborides (TMB_2_) were studied. To avoid human errors, the calculations were semi-automated prepared and analyzed for all TM-Borides using python- and bash-scripting, respectively. 28 transition metals were considered, including all 3d, 4d and 5d elements except for Mercury (In case of Re the calculation of elastic constants for the *α*- and *ω*-structure exceeded the chosen time limit for computational effort as the compound was studied in detail in literature^18,19^). Therefore, all energy cutoffs and k-point meshes, where carefully chosen to ensure energy convergence of a few meV/at. The lattice parameter, the equilibrium volume and bulk moduli where fitted using a Birch-Murnaghan fit. To calculate the single-crystal elastic constants the method suggested by R. Yu *et al*.^48^ was obtained. Furthermore, the formation energy of single vacancies where studied using the Alloy-Theoretic Automated Toolkit^49^ where in the case of *α*-structure 3 × 3 × 3 supercells containing 81 Atoms and 3 × 3 × 1 supercells containing 108 atoms for the *ω*-structure where created. For the *α*-structure one - either metal or boron site - was eliminated which corresponds to a vacancy concentration of ~3.7 at.% and ~1.9 at.%, respectively. However, concerning the *ω*-structure, the two different boron sites (flat and puckered) as well as the metal site where taken into account interrelated to ~1.4 at.% boron vacancies and ~2.8 at.% metal vacancies.

### Data availability

The datasets generated and analysed during the current study are available from the corresponding author on reasonable request.

## Electronic supplementary material

Supplementary Information
