# Supplementary material for: Ab initio inspired design of ternary boride thin films
Source: Sci Rep. 2018 Jun 18;8:9288. doi: 10.1038/s41598-018-27426-w (PMC6006173; doi:10.1038/s41598-018-27426-w)
Supplement: Supplementary file 1 — Supplementary Information [file 41598_2018_27426_MOESM1_ESM.pdf]

Ab initio inspired design of ternary boride thin films

Vincent Moraes,[1,*] Helmut Riedl,[1,2] Christoph Fuger[1], Peter Polcik[3], Hamid Bolvardi[4], David Holec[1], Paul Heinz Mayrhofer[1,2]

[1]Christian Doppler Laboratory for Application Oriented Coating Development at the Institute of Materials Science and Technology, TU Wien, A-1060 Wien, Austria

[2]Institute of Materials Science and Technology, TU Wien, A-1060 Wien, Austria

[3]Plansee Composite Materials GmbH, D-86983 Lechbruck am See, Germany

[4]Oerlikon Balzers, Oerlikon Balzers Surface Solutions AG, FL-9496 Balzers, Liechtenstein

[*]E-mail: vincent.moraes@tuwien.ac.at

Appendix

Table S.1: Ground state properties: lattice constants, bulk moduli, shear moduli, and density of TMB_2 in the α (SG-191) and ω (SG-194) structure, respectively.

XB_2	Structure type	a [Å]	b [Å]	c [Å]	B_{EC} [GPa]	B_{BM} [GPa]	G [GPa]	density [g/cm^3]
AgB_2	α	3.035	3.035	4.081	81	134	-42	6.6
	ω	3.017	3.017	17.011	66	85	-9	6.4
AuB_2	α	3.007	3.007	4.351	-41	127	-11	10.7
	ω	3.007	3.007	17.901	78	75	33	10.4
CdB_2	α	3.128	3.128	4.097	75	112	13	6.4
	ω	3.076	3.076	18.269	61	71	-1	5.9
CoB_2	α	3.006	3.006	2.809	203	245	70	6.1
	ω	3.005	3.005	11.660	251	226	180	5.9
CrB_2	α	2.970	2.970	3.003	202	287	143	5.3
	ω	2.912	2.912	12.845	252	271	175	5.2
CuB_2	α	2.985	2.985	3.403	85	167	33	5.4
	ω	2.977	2.977	13.947	43	154	-20	5.3
FeB_2	α	3.025	3.025	2.822	125	185	56	5.8
	ω	2.956	2.956	12.248	221	234	115	5.6
HfB_2	α	3.147	3.147	3.491	230	250	187	11.1
	ω	3.139	3.139	15.330	195	185	106	10.2
IrB_2	α	3.123	3.123	3.227	174	259	35	13.0
	ω	2.978	2.978	15.046	-1568	173	20	12.3
MnB_2	α	2.992	2.992	2.853	174	266	68	5.8
	ω	2.931	2.931	12.327	261	270	129	5.5
MoB_2	α	3.033	3.033	3.327	292	307	124	7.4
	ω	3.022	3.022	13.971	279	305	165	7.1
NbB_2	α	3.113	3.113	3.324	277	282	149	6.8
	ω	3.058	3.058	14.689	252	245	177	6.4
NiB_2	α	2.996	2.996	3.054	67	203	3	5.6
	ω	2.955	2.955	12.688	177	190	63	5.6
OsB_2	α	2.986	2.986	3.439	239	299	204	13.2
	ω	2.931	2.931	14.945	286	218	-70	12.6
PdB_2	α	3.047	3.047	3.609	-5	176	-27	7.3
	ω	3.025	3.025	14.740	119	153	23	7.3
PtB_2	α	3.044	3.044	3.714	-123	167	-62	12.1
	ω	3.125	3.125	14.489	-59	145	11	11.7
ReB_2	α	3.098	3.098	3.170	-	329	-	13.9
	ω	2.984	2.984	14.045	-	333	-	12.8
RhB_2	α	3.088	3.088	3.222	201	263	38	7.8
	ω	2.977	2.977	14.729	-98	191	-51	7.3
RuB_2	α	3.028	3.028	3.290	204	286	28	7.8
	ω	2.967	2.967	14.262	64	227	-33	7.5
ScB_2	α	3.147	3.147	3.532	171	189	152	3.7
	ω	3.137	3.137	15.427	29	131	84	3.4
TaB_2	α	3.103	3.103	3.331	287	292	144	12.1
	ω	3.053	3.053	14.650	254	277	176	11.4
TcB_2	α	2.971	2.971	3.419	252	292	86	7.6
	ω	2.991	2.991	13.855	270	295	121	7.4
TiB_2	α	3.035	3.035	3.229	229	247	195	4.5
	ω	3.016	3.016	14.293	228	232	153	4.1
VB_2	α	3.000	3.000	3.032	253	279	179	5.1
	ω	2.941	2.941	13.435	238	261	169	4.8
WB_2	α	3.024	3.024	3.380	310	309	109	12.7
	ω	3.025	3.025	14.072	347	326	213	12.2
YB_2	α	3.302	3.302	3.867	147	157	113	5.0
	ω	3.238	3.238	17.126	35	127	146	4.7
ZnB_2	α	3.058	3.058	3.397	121	132	32	5.2
	ω	3.065	3.065	14.308	76	129	49	5.0
ZrB_2	α	3.181	3.181	3.557	218	235	174	6.0
	ω	3.161	3.161	15.634	180	195	83	5.5

Table S.2: Elastic constants of tranistion metal diborides in the corresponding α- and ω-type structure, respectively.

XB_2	Structure type	C_{11} [GPa]	C_{12} [GPa]	C_{13} [GPa]	C_{33} [GPa]	C_{44} [GPa]	C_{66} [GPa]
AgB_2	α	249	180	38	194	-21	35
	ω	138	250	-19	362	-25	-56
AuB_2	α	218	226	66	71	-16	-4
	ω	256	130	-4	193	20	63
CdB_2	α	234	123	74	105	8	55
	ω	214	115	18	123	-12	49
CoB_2	α	515	210	131	286	61	153
	ω	474	180	132	538	170	147
CrB_2	α	563	135	57	366	163	214
	ω	572	115	84	591	230	229
CuB_2	α	305	246	48	221	-28	29
	ω	115	421	-50	466	-30	-153
FeB_2	α	441	201	158	136	101	120
	ω	486	139	129	306	173	174
HfB_2	α	591	50	88	412	261	271
	ω	405	102	81	449	120	152
IrB_2	α	367	265	181	313	-68	46
	ω	239	322	125	0	29	-41
MnB_2	α	457	233	211	182	155	112
	ω	475	154	155	470	209	160
MoB_2	α	599	123	167	410	138	238
	ω	567	117	127	620	221	225
NbB_2	α	570	97	158	370	217	236
	ω	544	117	97	581	239	213
NiB_2	α	317	301	104	218	-47	8
	ω	396	152	111	260	68	122
OsB_2	α	482	269	130	500	-119	106
	ω	374	70	579	-171	-42	152
PdB_2	α	134	349	29	216	-24	-107
	ω	307	164	71	215	1	71
PtB_2	α	150	323	55	89	-98	-87
	ω	256	182	-12	-40	-1	37
RhB_2	α	447	205	125	337	14	121
	ω	86	352	26	131	-56	-133
RuB_2	α	419	252	161	350	16	83
	ω	261	307	142	252	-99	-23
ScB_2	α	492	35	41	342	185	228
	ω	344	43	82	0	38	150
TaB_2	α	578	125	169	385	209	226
	ω	558	115	95	586	247	221
TcB_2	α	570	179	118	460	52	195
	ω	526	136	139	548	135	195
TiB_2	α	638	59	75	392	256	289
	ω	491	138	87	527	156	176
VB_2	α	662	109	94	426	223	277
	ω	538	89	92	514	236	225
WB_2	α	596	143	194	365	121	226
	ω	651	170	190	680	252	240
YB_2	α	353	49	52	312	157	152
	ω	312	30	-5	0	99	141
ZnB_2	α	358	157	40	229	1	100
	ω	289	239	-104	0	0	25
ZrB_2	α	548	43	89	384	246	252
	ω	359	100	81	414	84	129
